# Effect of Solar Radiation on Skin Microbiome: Study of Two Populations

**DOI:** 10.3390/microorganisms10081523

**Published:** 2022-07-27

**Authors:** Nurit Harel, Leah Reshef, Dvora Biran, Sarah Brenner, Eliora Z. Ron, Uri Gophna

**Affiliations:** 1 The Shmunis School of Biomedicine and Cancer Research, Faculty of Life Sciences, Tel Aviv University, Tel Aviv 69978, Israel; nuritharel@mail.tau.ac.il (N.H.); leahfa@gmail.com (L.R.); birand@tauex.tau.ac.il (D.B.); urigo@tauex.tau.ac.il (U.G.); 2 Porter School of the Environment and Earth Sciences, Tel Aviv University, Tel Aviv 69978, Israel; 3 Sackler Faculty of Medicine, Tel Aviv University, Tel Aviv 69978, Israel; sarahb@post.tau.ac.il

**Keywords:** skin microbiome, sun radiation, human microbiome

## Abstract

Here, we examined the skin microbiome of two groups of healthy volunteers living on the Mediterranean coast with different exposures to sun radiation. One group, exposed to the sun in the summer, was compared with a group covered with clothing throughout the year. The seasonal effects on the skin microbiome of three body sites were determined before and after summer. Surprisingly, at the phyla level, there were no significant differences in microbiome diversity between the groups. Furthermore, within each group, there were no significant seasonal differences in high-abundance species at any of the sampling sites. These results suggest that the skin microbiome, developed over years, remains stable even after several months of exposure to summer weather, direct sunlight and humidity. However, in the group exposed to the sun during the summer months, there were significant differences in low-abundance species in sun-exposed areas of the skin (the inner and outer arm). These subtle changes in low-abundance species are interesting, and their effect on skin physiology should be studied further.

## 1. Introduction

Human skin is regularly subjected to environmental influences that cause stress. Excessive sun exposure is one of the major environmental factors which contribute to skin damage, as solar radiation can cause DNA damage and long-term effects. Consequently, the skin utilizes several endogenous mechanisms in order to deal with this threat. Some of the known mechanisms include increasing epidermal thickness, DNA repair mechanisms (NER and BER), apoptosis, antioxidant production, enzyme production and skin pigmentation. The skin microbiome may well be an additional protective mechanism, and it is reasonable to assume that the relationship between microbiomes and photoprotection is more complex than previously assumed. The symbiotic microorganisms in the skin [1] occupy a variety of skin niches and may act as protective agents against the invasion of more pathogenic or harmful organisms, supporting the barrier activity of the skin both physically and immunologically [2]. According to recent studies, skin microbes regulate gene expression in the skin [3] and influence its immune response [4]. Thus, *Propionibacterium acnes*, *Staphylococcus epidermis*, *Staphylococcus aureus*, *Corynebacterium diphtheria*, *Corynebacterium jeikeium* and *Pseudomonas aeruginosa,* which are part of the skin microbiome, are assumed to contribute to the health of the skin. For example, *S. epidermidis*, isolated from healthy skin, produces a number of antimicrobial compounds that inhibit the formation of biofilms by pathogenic bacteria [5,6]. The microbiome receives and is affected by the same exposure to solar radiation as the skin [7]. The size of the bacteria prevents them from developing efficient photoprotection against solar radiation [8], and their genetic material comprises a significant portion of their cellular volume [9], making bacteria among the most vulnerable to photodamage. Indeed, field studies on marine bacteria have demonstrated a decrease in total bacterial abundance, amino acid uptake [10], exoenzymatic activity [11] and oxygen consumption [12].

Here, we examined the impact of direct exposure to sun irradiation on the skin microbiome of healthy volunteers from two groups living in the same geographic area (Mediterranean coast). We examined the skin microbiome on three body sites (the cheek, inner arm and outer arm) before and after summer in a sun-exposed group (lifeguards) compared with a sun-protected population (ultraorthodox). As the group of sun-protected volunteers constituted of people who wore heavy clothes year-round, the data obtained in this study provided additional information on the effect of lifestyle on the skin microbiome.

## 2. Materials and Methods

### 2.1. Study Population

Two groups of healthy male volunteers of approximately the same age were studied: lifeguards exposed to high levels of solar radiation over the years and ultraorthodox sun-protected all year-round by heavy clothing.

Both groups were exposed to the same characteristics of summer weather, including sun radiation, humidity and high temperature. One group was composed of lifeguards, exposed yearly to direct sunlight and seawater throughout summer. The second group consisted of ultraorthodox, who wore long sleeves and were protected from direct sunlight at all times.

In total, 122 samples were collected for microbiome analysis, consisting of 66 samples of male lifeguards (36 before and 30 after summer) and 56 samples of ultraorthodox males (30 before and 26 after summer). For mycobiome analysis, 116 samples were collected, consisting of 62 samples of lifeguards (34 before and 28 after summer) and 54 samples of the ultraorthodox (28 before and 26 after summer). The data are summarized in Table 1.

### 2.2. Sampling

Three skin sites were sampled: the inner forearm, dorsal forearm and cheek. The first sampling was conducted before the summer and enabled the comparison of microbiome diversity following years of exposure to solar radiation versus skin protected by heavy clothing. This microbiome population was called the “baseline”.

The second sampling was conducted at the end of the summer. This study provided data on the effect of the sun and summer conditions on the skin microbiome.

Noninvasive swabs (FLOQSwab hgDNAfree appl, 2502CS01, Copan, Brescia, Italy) were used to collect skin microbes. The swabs were soaked in sterile phosphate-buffered solution with pH 7.2 and 0.1% polysorbate 80 (extraction fluid) before they were rubbed against the skin for 30 s. After sampling, the swabs were immediately cooled and frozen.

Sample collection was approved by the Laniado Hospital Helsinki committee and all subjects provided informed consent.

### 2.3. DNA Extraction and PCR Amplification

Total microbial DNA was isolated from the skin swab samples according to the HMP (Human Microbiome Project) guidelines, using the DNeasy PowerSoil Kit (QIAGEN, Hilden, Germany, 12888).

Bacterial PCR amplification of the 16S rRNA gene was carried out with universal prokaryotic primers containing 5’-end common sequences (CS1-341F 5’-CACTGACGACATGGTTCTACANNNNCCTACGGGAGGCAGCAG and CS2-806R 5′-TACGGTAGCAGAGACTTGGTCTGGACTACHVGGGTWTCTAAT). Thirty PCR cycles (95 °C for 15 s, 53 °C for 15 s, 72 °C for 15 s) were conducted using the PCR Mastermix KAPA2G Fast™ (KAPA Biosystems, Wilmington, MA, USA) and successful amplification was verified with agarose gel electrophoresis. For fungal analysis, the ITS2 region of the rDNA operon was amplified using primers 3271-ITS2F: GTGARTCATCGAATCTTT and 3271-ITS2R: GATATGCTTAAGTTCAGCGGGT, adapted from [13,14].

For sequencing, Illumina adaptors and sample-specific barcodes (San Diego, CA, USA) were added in a 2nd 8-cycle PCR, which targeted the CS1/CS2 common linkers, performed at the sDNA Services (DNAS) facility within the Research Resources Center (RRC) at the University of Illinois in Chicago (UIC). Library products were purified using AMPure beads, quantified, pooled and paired-end sequenced (2 × 250) using the Illumina MiSeq platform [15].

Sequencing depth ranged from 1474 to 34,000 sequences per sample. The data were rarefied to 1400 seqs/sample to ensure data evenness.

### 2.4. Data Analysis

Raw data were demultiplexed and trimmed of adaptors at the sequencing center. A custom R script was then used to remove sequencing primers and trim readthrough events (common in ITS sequencing due to the variable length of the ITS region). The DADA2 R package [16] was then used for quality control, inference of exact sequences, detection and removal of chimeric sequences and taxonomic assignment against Silva database version 132 (for 16S) or UNITE ITS database versions 02.02.2019 (for ITS). Data were ratified to an even depth of 1400 seq/sample [17,18].

Principle coordinate analysis (PcoA) and analysis of similarity (ANOSIM) were conducted in R using the vegan package [19].

The LefSe [20] algorithm was applied to identify which bacterial taxa contributed to the differences between the two groups.

## 3. Results

### 3.1. The Microbiomes of Lifeguards and Ultraorthodox Males Are Broadly Similar

The purpose of the study was to examine the effect of summer exposures, including strong solar radiation, on the microbiome and mycobiome of two groups of people living along the Mediterranean coast.

**Figure 1:**
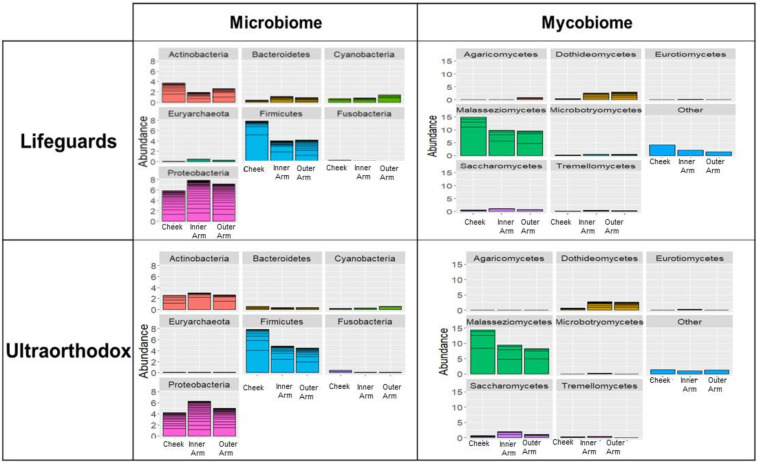
An analysis of OTU abundance by phylum. A comparison of the microbiomes and mycobiome of the two groups—mean relative abundance (>1%), at least 10% of reads in at least one sample.

The microbiome samples of each group were analyzed, and the mean relative abundances were calculated, focusing on phyla with >1% relative abundance. The results shown in Figure 1 indicate that there were minor differences between lifeguards and ultraorthodox at the phylum level, both in terms of the microbiome and mycobiome (Figure 1). Proteobacteria and Firmicutes were the most dominant bacterial phyla in both groups, followed by Actinobacteria. In the mycobiome analysis, Malasseziomycetes were the most dominant class in both groups, followed by Dothideomycetes and Saccharomycetes. Similar microbial/mycobial prevalences across the other body sites were observed for both subject groups, with the cheek showing enrichment in Firmicutes and Malasseziomycetes, and a reduction in Proteobacteria and Dothideomycetes, as compared to the arm.

**Figure 2:**
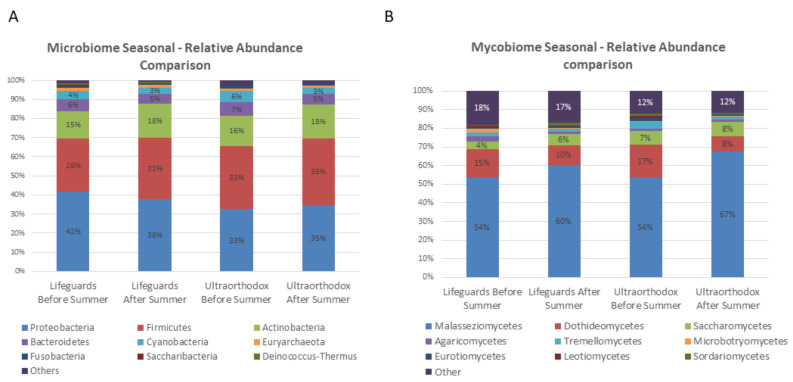
Seasonal comparison of mean relative abundance in microbiomes between lifeguards and ultraorthodox (>1%). (**A**) microbiome; (**B**) mycobiome.

The bacterial and fungal microbiomes of each group, before and after summer, were compared, focusing on phyla with >1% relative abundance (Figure 2). No major seasonal effects were observed in any study groups based on the Wilcoxon signed ranks test.

Within the mycobiome (fungal microbiome), Malasseziomycetes were the most dominant phylum both before and after summer, followed by Dothideomycetes and Saccharomycetes.

### 3.2. Microbial Diversity Is Maintained after the Summer Season in Both Lifeguards and Ultraorthodox

**Figure 3:**
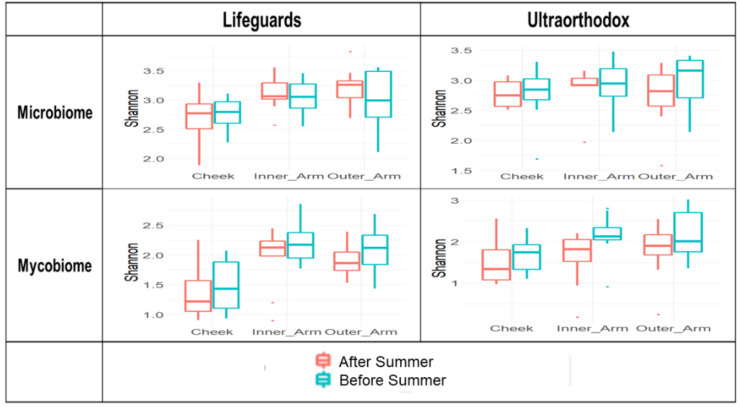
Alpha diversity of skin microbiome across body sites and subject groups, before and after summer. Boxplots depict medians, with lower and upper bounds showing 1st and 3rd quartiles, respectively; outliers shown as points.

The microbiomes of the two groups were of similar diversity (Figure 3), measured with the Shannon index, which considers both richness (the number of taxonomic groups) and evenness (the distribution of abundances within the groups), across all sampled body sites. Following the summer exposures, there were no changes in the Shannon diversity that reached statistical significance in either group, with the exception of a slight increase in fungal diversity in the inner arm samples from the ultraorthodox group (Kruskal–Wallis test, *p* = 0.04).

### 3.3. The Microbial Composition of the Lifeguards’ Changes Following Summer

To explore in-depth seasonal changes occurring in each group, genus-level data were transformed into distance matrices using either Jaccard or Bray–Curtis similarity indices, and principal coordinate analysis (PcoA) coupled with ANOSIM significance testing was then applied. The Bray–Curtis coefficient incorporates both the presence and abundance of taxa, thus, giving a greater weight to species with high abundance, while the Jaccard index is based exclusively on the presence/absence of genera, thereby giving similar weight to highly abundant and rare taxa.

When comparing the groups in terms of their microbiome composition at the genus level in the three skin sites before summer, no statistically significant differences were observed using either the Jaccard or Bray–Curtis indices of similarity with the ANOSIM test (for the principle coordinate analysis, see Table 2). Before summer, the only statistically significant difference was observed at the cheek site using the Jaccard index (*p* = 0.021).

**Figure 4:**
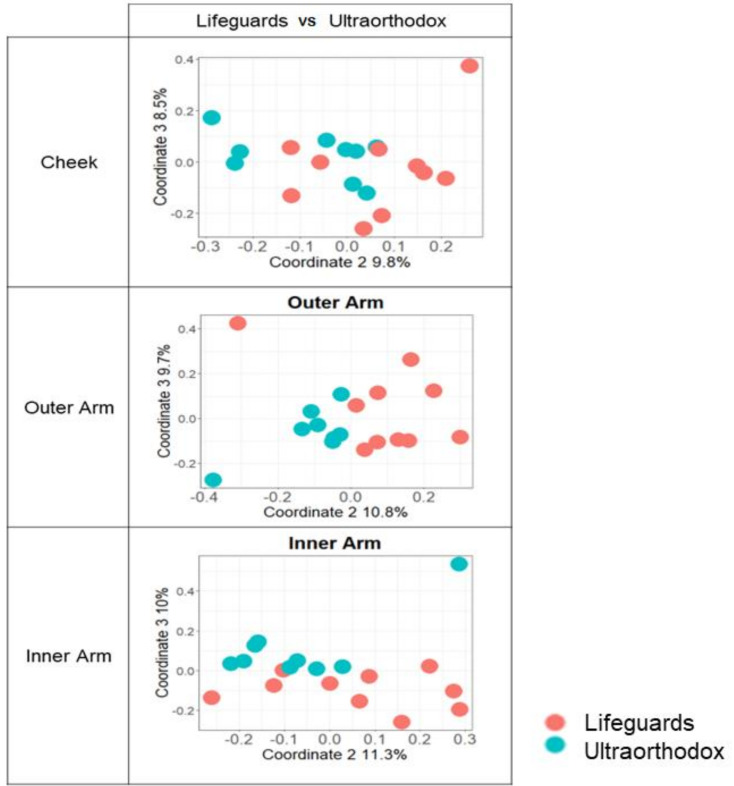
Beta diversity analysis of the microbiome of lifeguards versus ultraorthodox before and after summer. PcoAs of Jaccard distance matrices are shown. Blue—ultraorthodox; orange—lifeguards.

However, following summer exposures, significant differences in microbial composition between lifeguards and ultraorthodox could be detected in all three sampling sites when using the Jaccard, but not Bray–Curtis, similarity index (Table 2, Figure 4). In accordance with this, using the Jaccard index, we also detected a weak but significant seasonal bacterial difference in the microbiome (bacterial) within the lifeguard group (*p* = 0.001, R = 0.13). In contrast, there was no significant seasonal difference in the microbiome within the ultraorthodox group (Table 3).

**Figure 5:**
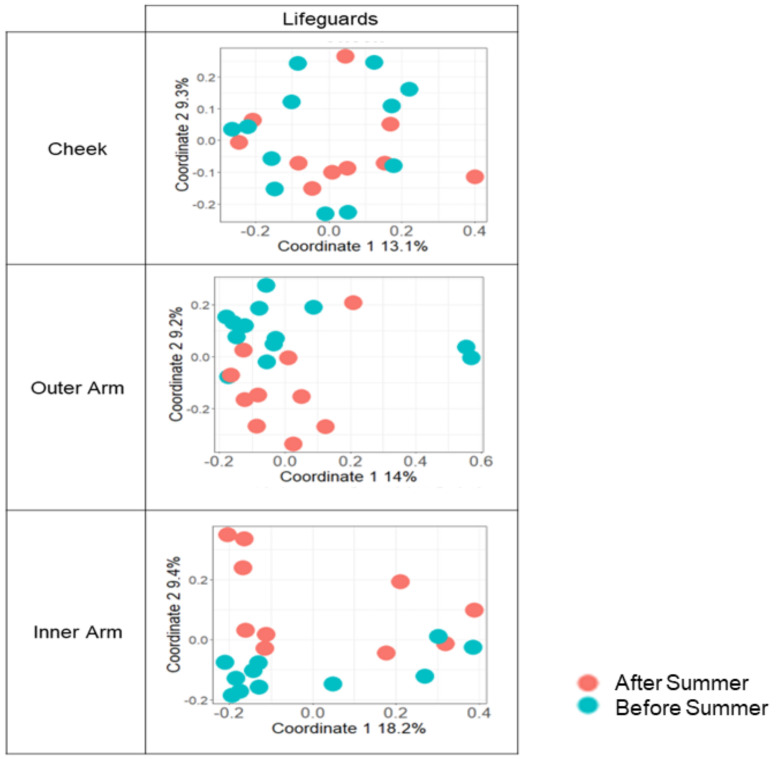
Seasonal differences of lifeguard’s skin microbiome across several body sites. PcoAs of Jaccard distance matrices are shown. Orange—after the summer; blue—before the summer. The percentage of variance is shown for each axis; ANOSIM p and R values were calculated for each facet and are shown in Table 4.

A site-specific analysis within the lifeguard group revealed that seasonal differences in the microbiome were being driven by the microbial communities of the outer and inner arm, which were the sites exposed to the sun in this population, often not protected by a hat or sunscreen (Table 4, Figure 5). In contrast, no significant seasonal differences were found in the mycobiomes of either site.

### 3.4. Specific Taxa Driving Seasonal Differences in the Lifeguards’ Microbiome

**Figure 6:**
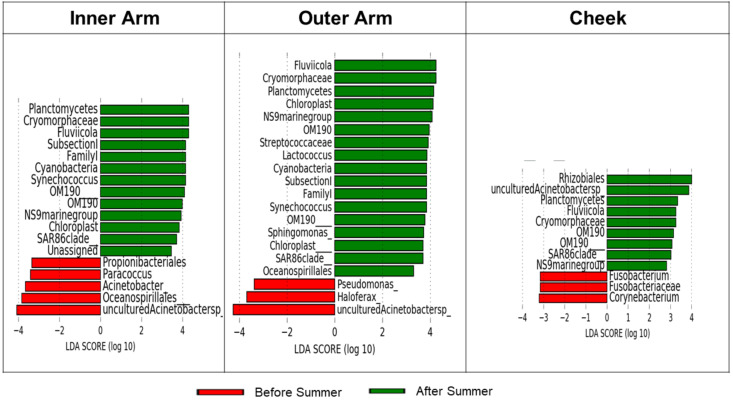
Microbial taxa driving seasonal differences in skin microbiome of lifeguards. Discriminating taxa were identified with LefSe; taxa with prevalence <0.25 were excluded from the analysis.

We next attempted to uncover which specific taxa drove the seasonal differences within the lifeguard group using LEfSe (linear discriminant analysis effect Size). Only 2–3% of the microbiome population was affected by seasonal changes, while 97% remained stable (Figure 6). Environmental bacteria, such as Planctomycetes, Cryomorphaceae, SAR 86, etc., drove most of the differences between the seasons. When these were excluded from the comparison, there were a few known human-associated taxa left that showed a difference between the seasons, e.g., Streptococcaceae [21] and Cyanobacteria [22], which were present only after summer.

## 4. Discussion

Here, we examined the fungal and bacterial skin microbiomes of two groups of individuals who live along the Mediterranean coast but have very different lifestyles. The first group consisted of lifeguards, an occupation with excessive exposure to sun radiation during summer over the years. The second group consisted of ultraorthodox Jews, who wear long-sleeved clothes throughout the year and, therefore, the exposure of their arms to the sun is minimal. Samples were taken from three body sites—the cheek, inner arm and outer arm.

Although very different in lifestyle, the skin microbiome (at the phylum level) of the two groups was broadly similar, showing a certain level of conservation of the human skin microbiome. Accordingly, an analysis of the skin microbiomes of the two groups at the start of the study indicated no statistically significant differences at the phylum levels in their bacterial or fungal microbiomes. Proteobacteria, Firmictus, and Actinobacteria composed over 85% of the bacterial population on the skin, while Malasseziomycetes, Dothideomycetes, and Saccharomyxetes accounted for over 65% of the fungal population. Thus, differences in lifestyle were not sufficient to generate phylum level differences between those groups.

Previous experiments comparing the microbiomes of groups with different lifestyles demonstrated differences in the skin microbiome community structure [23]. However, the comparison was between Pakistani and Chinese students, so the differences may very well reflect the genetic variation between the groups. Indeed, additional studies [24] showed that the microbial composition of Chinese people was different from that of other ethnic groups. In our study, all the volunteers were of the same ethnic group but differed in lifestyle. Therefore, we conclude that lifestyle had a minimal effect on the basic microbiome composition.

A study by Burns et al. followed the skin microbiome 24 h after a single exposure to UVB and UVA. This study also indicated that there was no significant change in the microbiome, except for an increase in the relative abundance of Cyanobacteria [18]. We studied the accumulated effects of daily exposure to sun radiation over several months, and even after long exposure, there were no significant changes in the highly abundant phyla. There were seasonal changes observed in the microbiome after the summer, but they were probably not primarily due to sun exposure, as they also occurred in the ultraorthodox.

The alpha diversity measurements of species diversity before and after the summer showed only one difference in the mycobiome of the inner hand of the ultraorthodox. This change could be due to the increased humidity in the inner arm of people wearing long sleeves on hot summer days. The results were consistent with a study that followed the skin microbiome over a period of two years in healthy volunteers. This study concluded that the skin microbiome remained stable, despite exposure to extreme environmental conditions [25]. This stability was maintained, although there were indications that seawater washed away some of the human microbiome [26].

Beta diversity analysis was used to assess compositional dissimilarities between the two groups. Two different distance indices were used: Bray–Curtis, which considers prevalence as well as relative abundance, giving a higher weight to species with higher relative abundance; and the Jaccard index, which is based only on presence/absence (all taxa have equal weights, regardless of their relative abundance). The data from all sampling points were analyzed for each individual according to the sampling time, and the changes in relative abundance before and after the summer were calculated separately for each of the two groups. According to the Bray–Curtis index, there was no significant difference in the microbiome or mycobiome composition in either group before and after summer. However, using the Jaccard index, we could show a significant change in the microbiomes of lifeguards after summer. This difference could be seen only in the lifeguards, suggesting that this microbial change could be traced to seawater exposure, while others may be due to exposure to sun radiation. The difference could be attributed to a change in the low-abundance species, as it could only be seen in the Jaccard index, which gives equal weight to rare species. Thus, it appeared that daily exposure to sun radiation during a five-month period resulted in a significant change in low-abundance species. The change in low-abundance species was consistent with findings that indicated that there is a difference in the sensitivity of bacteria to radiation. It was shown that prolonged and intense exposure to UV-R is selectively tolerated by some bacteria and fungi, but not by others [27].

The changes of the microbiome following exposure to sun radiation could be observed only in the inner and outer arm. This finding indicated that the change was due to exposure to the sun, rather than exposure to other conditions such as sea spray. Unexpected was the finding that there was no significant difference in the cheek microbiome. One possible explanation is that the lifeguards protected their face with wide-brimmed hats and sunscreen, and paid more attention to their facial hygiene, but were less concerned with protecting their arms. Thus, the lifeguard’s inner and outer arms were exposed to the direct radiation of the summer sun, leading to changes in the composition of low-abundance species.

In medicine, bacterial–fungal interactions are of great importance. There is evidence that bacterial factors may influence fungal growth or physiology, and that fungi, in turn, may influence bacterial behavior and survival. Bacteria and fungi can interact in a variety of ways, including direct contact between the cells, chemical interactions, such as the secretion of quorum-sensing molecules, and changes in the host’s response [28]. In this study, we found that the majority of the microbiome of healthy skin (bacterial and fungal) remained stable even after insolation. This raised the question into how bacteria and fungi and their metabolites interact throughout the year, facilitating their ability to adapt to hot, humid summer climate conditions.

Certain biases are inherent to the NGS of marker genes, used here to infer microbial composition. These arise from differences in the copy number of ribosomal operons across species and in primer specificity, as well as from stochastic compositional changes brought on by multiple PCR cycles. A universal protocol was applied across all samples in this study to allow for a between-sample comparison. Species-level resolution was difficult to infer in 16S rRNA-based NGS pipelines, as short (~350 bp) fragments may have had an equally good match to several species. Additionally, 16S, and more so ITS, databases do not yet represent the whole microbial biodiversity of marine environments. Thus, only 3.1% of the ASVs in our data was classified to a single species.

## 5. Conclusions

The study of the microbiome in health is important for understanding the relationship between the microbiome and the normal function of the skin. Based on the results of this study, we found that climatic conditions had a larger impact on the composition of the human skin microbiome and mycobiome than living habits. Interestingly, two groups of people with differing lifestyle habits and different sun exposure habits exhibited a broadly similar microbiome composition.

Based on our results, one may speculate that the seasonal effects on the microbiome, regardless of whether the living habits of the subjects are associated with high sun exposure or low sun exposure, are generally tolerable by most members of the skin microbiome that have evolutionarily adapted to such changes. This could be the reason for the fact that the majority of the human skin microbiome remained stable even when exposed to sun and seawater on a daily basis for months. The difference in the low-abundance species was interesting and should be explored further. In addition, future studies should explore the secreted metabolome of skin microorganisms across different seasons, and its effects on the human host.

## Figures and Tables

**Table 1:** Summary of the samples collected.

	**Microbiome**			
	**Outer Arm**	**Inner Arm**	**Cheek**	
Lifeguards	10	10	10	After Summer
	13	11	12	Before Summer
Ultraorthodox	8	9	9	After Summer
	11	11	10	Before Summer
	**Mycobiome**			
	Outer Arm	Inner Arm	Cheek	
Lifeguards	9	9	10	After Summer
	11	11	12	Before Summer
Ultraorthodox	8	9	9	After Summer
	8	10	10	Before Summer

**Table 2:** Beta diversity analysis of the microbiome of the lifeguards versus ultraorthodox before and after summer. Between-group dissimilarities were calculated using Jaccard and Bray–Curtis distances. Bold indicates significant difference.

	Beta Diversity—Lifeguards vs. Ultraorthodox							
	Before Summer				After Summer			
	Bray–Curtis		Jaccard		Bray–Curtis		Jaccard	
	*p* Value	R	*p* Value	R	*p* Value	R	*p* Value	R
Cheek	0.119	0.07	**0.021**	0.17	0.133	0.08	**0.005**	0.23
Outer Arm	0.669	0.04	0.738	0.05	0.089	0.11	**0.039**	0.14
Inner Arm	0.4	0	0.32	0.02	0.085	0.11	**0.039**	0.14

**Table 3:** Seasonal effects on the microbiome and mycobiome. Microbiome and mycobiome dissimilarities were calculated for each group using Jaccard and Bray–Curtis distances. Bold indicates significant difference.

	Microbiome				Mycobiome			
	Bray–Curtis		Jaccard		Bray–Curtis		Jaccard	
	*p* Value	R	*p* Value	R	*p* Value	R	*p* Value	R
**Lifeguards**	0.07	0.03	**0.001**	0.13	0.341	0.01	0.323	0.01
**Ultraorthodox**	0.107	0.03	0.399	0	0.397	0	0.061	0.03

**Table 4:** Seasonal effect on skin microbiome of several body sites of lifeguards. Beta diversity analysis was performed for each body site before and after summer. Between-season dissimilarities were calculated using Jaccard and Bray–Curtis distances. Bold indicates significant differences.

	Bray–Curtis		Jaccard	
	*p* Value	R	*p* Value	R
Cheek	0.595	0.08	0.215	0.05
Outer Arm	0.495	0.01	**0.044**	0.12
Inner Arm	0.209	0.04	**0.058**	0.12

## Data Availability

Not applicable.
